# Adsorption Behavior of Crystal Violet and Congo Red Dyes on Heat-Treated Brazilian Palygorskite: Kinetic, Isothermal and Thermodynamic Studies

**DOI:** 10.3390/ma14195688

**Published:** 2021-09-30

**Authors:** Vanderlane Cavalcanti Silva, Maria Eduarda Barbosa Araújo, Alisson Mendes Rodrigues, Maria do Bom Conselho Vitorino, Juliana Melo Cartaxo, Romualdo Rodrigues Menezes, Gelmires Araújo Neves

**Affiliations:** 1 Graduate Program in Materials Science and Engineering (PPG-CEMat), Federal University of Campina Grande, Av. Aprígio Veloso-882, Bodocongó, Campina Grande 58429-900, PB, Brazil; vanderlanecavalcanti@outlook.com (V.C.S.); mariaeduardaba@hotmail.com (M.E.B.A.); adrsames@gmail.com (M.d.B.C.V.); 2 Laboratory of Materials Technology (LTM), Department of Materials Engineering, Federal University of Campina Grande, Av. Aprígio Veloso-882, Bodocongó, Campina Grande 58429-900, PB, Brazil; julianamelo25@gmail.com (J.M.C.); romualdo.menezes@ufcg.edu.br (R.R.M.); gelmires.neves@ufcg.edu.br (G.A.N.)

**Keywords:** palygorskite, adsorption, crystal violet, congo red, water treatment

## Abstract

The effect of heat treatment on the adsorptive capacity of a Brazilian palygorskite to remove the dyes crystal violet (CV) and congo red (CR) was investigated. The natural palygorskite was calcined at different temperatures (300, 500 and 700 °C) for 4 h. Changes in the palygorskite structure were evaluated using X-ray diffraction, X-ray fluorescence, thermogravimetric and differential thermal analysis, N_2_ adsorption/desorption and Fourier transform infrared spectroscopy. The adsorption efficiency of CV and CR was investigated through the effect of initial concentration, contact time, temperature, pH and dosage of adsorbent. The calcination increased the adsorption capacity of palygorskite, and the greatest adsorption capacity of CV and CR dyes occurred in the sample calcined at 700 °C (Pal-700T). The natural and calcined samples at 300 and 500 °C followed the Freundlich isothermal model, while the Pal-700T followed the Langmuir isothermal model. Adsorption kinetics results were well described by the Elovich model. Pal-700T showed better adsorption performance at basic pH, with removal greater than 98%, for both dyes. Pal-700T proved to be a great candidate for removing cationic and anionic dyes present in water.

## 1. Introduction

Water pollution due to the effluents discharged daily by various industries, such as textiles, pharmaceuticals, paper, plastics and cosmetics, is considered one of the biggest environmental problems in the world [1,2]. Effluents derived from these industries are often rich in dyes [3,4]. Dye pollution is a threat to human health and aquatic ecosystems since most of them are highly toxic, mutagenic, allergenic and carcinogenic, and can quickly accumulate in living cells, harming an entire food chain [5,6,7].

Crystal violet (CV) is a triphenylmethane cationic dye commonly used in the textile, paper and medical industries [8,9]. CV is considered a biohazardous substance due to its highly genotoxic, toxic, mutagenic and carcinogenic nature [10,11,12]. In addition, CV has a very intense color, and its presence in the aquatic environment, even at low concentrations (for example, 1 mg/L), increases water turbidity, making photosynthesis by aquatic plants impossible [12]. Human exposure to CV can cause eye irritation, increased heart rate, permanent blindness, respiratory disease, kidney failure, chemical cystitis and cancer [13,14,15,16,17].

Congo red (CR) is a diazo anionic dye used primarily in the textile and paper industries [18,19]. The effluent contaminated with CR reduces the oxygen levels in the water, suffocating the aquatic flora and fauna [20]. CR is considered the most harmful and used dye in the world; its degradation can generate amine, benzidine and other potentially carcinogenic species [21,22,23]. In humans, CR can cause general weakness, gastrointestinal irritation, anorexia, mutation, lung and bladder cancers [24,25,26,27,28]. CV and CR dyes have a complex structure, are highly stable to light and heat and are difficult to degrade biologically [29,30]. Therefore, the separation of CV and CR dyes from the water has become a great challenge, which has motivated the search for efficient techniques to solve or minimize the environmental pollution caused [29,31,32,33].

Numerous techniques have been developed to remove dyes from wastewater, such as coagulation/flocculation [34], ion exchange [35], membrane filtration [36], oxidation [37], electrochemical degradation [38] and adsorption [39]. Among these, adsorption is one of the most attractive techniques due to its versatility, simplicity, ecologically correct and high efficiency [2,40,41,42,43]. Some adsorbent materials can have a high cost, such as activated carbon [44,45]. This motivated the search for alternative adsorbents with high efficiency and economically viable, such as zeolites [46], clays [47] and biomass [48].

Clays are often used as adsorbents for economic feasibility and environmental importance due to their low cost, abundance, immediate availability, non-toxicity and high adsorptive properties [49,50,51]. Due to its fibrous structure and high surface area, the palygorskite clay has gained considerable prominence as an adsorbent to remove various types of pollutants [44,45,52,53,54,55,56].

Several physical and chemical modifications are used in palygorskite to improve its adsorption properties, mainly acid activation [44,45,50] and surface modification [57,58,59]. However, few studies have addressed how heat treatment can influence palygorskite structure and adsorption properties [60,61,62]. Furthermore, little has been explored about how heat treatment can affect the structure of palygorskite found in Brazil. Thus, this work aimed to analyze the effect of heat treatment on Brazilian palygorskite’s structure and evaluate its potential in treating contaminated water with crystal violet and congo red dyes.

## 2. Materials and Methods

### 2.1. Raw Materials

Palygorskite clay was supplied by União Brasileira de Mineração S.A. (UBM, Soledade, PB, Brazil) with a particle size of 0.074 mm. The crystal violet (CV) and Congo red (CR) dyes were purchased from Synth (Diadema, SP, Brazil) and Dinâmica Química (Indaiatuba, SP, Brazil), respectively. Hydrochloric acid and ammonium hydroxide were purchased from VETEC (Duque de Caxias, RJ, Brazil).

### 2.2. Heat Treatment 

The heat treatment protocol involved three steps: (i) the samples were heated (5 °C/min) from room temperature to the heat treatment temperatures (300, 500 and 700 °C), (ii) 4-h isothermal treatment was carried out and (iii) the oven was turned off and then cooled to room temperature. The heat treatment was carried out in a muffle furnace (mod. 3000, EDG, São Carlos, SP, Brazil). The samples treated at 300 °C, 500 °C and 700 °C were named Pal-300T, Pal-500T and Pal-700T, respectively. The sample without heat treatment was called Pal.

### 2.3. Characterizations

X-ray diffraction (XRD-6000, Shimadzu, Kyoto, Japan) was performed using CuKα (λ = 1.54 Å), operated at 40 kV and 30 mA, in 2θ angular range of 5–50° and 0.02° of step size [63,64]. Chemical analysis was determined using X-ray fluorescence spectrometry (EDX-720, Shimadzu, Kyoto, Japan). Infrared spectra with Fourier transform (FTIR) were recorded in the spectral range from 4000 cm^−1^ to 400 cm^−1^, with 32 scans and 4 cm^−1^ resolutions, using KBr pellets (Vertex-70, Bruker, Billerica, MA, USA). Thermogravimetric (TGA) and differential thermal analysis (DTA) were performed under air atmosphere, with a heating rate of 10 °C/min (DTG-60H, Shimadzu, Kyoto, Japan). The average pore diameter and surface area were determined by nitrogen adsorption measurements at 77 K using an Auto-sorb iQ Station 1 analyzer (Anton Paar, Graz, Austria). The Brunner–Emmett–Teller (BET) method [65,66] was used to calculate the surface area. All nitrogen sorption data were analyzed using the Quantachrome^®^ ASiQwin™ software (Anton Paar, Graz, Austria).

### 2.4. Batch Adsorption Experiments

The effects of contact time, initial dye concentration, pH, amount of adsorbent and temperature were studied in the adsorption of CV and CR dyes. During the experiment, one parameter was varied while the other parameters remained constant. The adsorption experiments were carried out in vials containing 20 mL of dye solutions. The adsorbent-solution systems were stirred (150 rpm) at 25 °C for up to 360 min. The parameters of the contact time, initial dye concentration, pH, dosage of adsorbent and temperature were analyzed in the range of 15–360 min, 2.5–200 mg/L, 3–11, 10–40 mg e 25–55 °C, respectively. After the adsorption process, the samples were centrifuged at 3600 rpm for 5 min. The concentrations of both dyes left in the solutions were determined from the measured absorbance values in the supernatant at 582.5 nm and 501 nm for λ_max_ of CV and CR, respectively. These experiments were performed in a UV spectrophotometer (UV-1800, Shimadzu). The equilibrium adsorbed amount (q_e_) of CV and CR and the removal percentage of both dyes (%R) were estimated using Equations (1) and (2):

q_e_ = [(C_o_ − C_e_)V]/m, (1)

%R = [(C_o_ − C_e_)/C_o_] × 100, (2)

where q_e_ (mg/g) is the adsorption capacity, C_o_ (mg/L) and C_e_ (mg/L) are the initial and equilibrium concentrations, respectively. V (L) is the volume of the solution and m (g) the mass of the palygorskite samples.

Ethanol was used as a desorption medium to remove dye particles adsorbed on the samples. Adsorbents loaded with CV and CR were desorbed using 60 mL of ethanol and stirred at 150 rpm for 1 h. Then, the adsorbents were filtered, washed with distilled water, dried, and used again for the subsequent adsorption-desorption cycles.

## 3. Results and Discussion

### 3.1. Characterization of Natural and Heat-Treated Palygorskite

**Figure 1:**
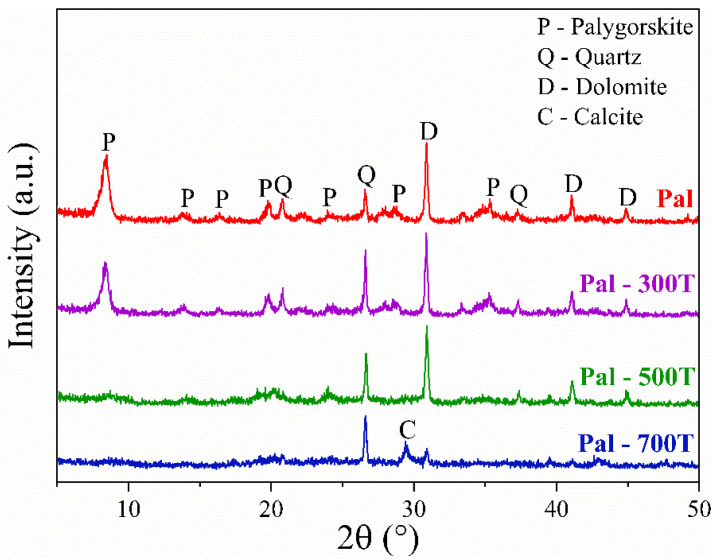
Palygorskite XRD patterns before and after heat treatment at different temperatures.

The X-ray diffraction patterns of natural and heat-treated palygorskite samples (300, 500 and 700 °C) are shown in Figure 1. As expected, the natural sample showed reflections of palygorskite (ICCD 21-0958), quartz (ICCD 46-1045) and dolomite (ICCD 36-0426) [45,67,68]. According to the heat treatment applied, different changes in the crystal structure were observed. For example, there was only a slight decrease in the characteristic reflections of palygorskite for the samples treated at 300 °C. Such behavior happened because there is a loss of zeolitic water at this temperature and a partial loss of coordinated water [69,70,71]. It is still possible to observe peaks characteristic of palygorskite in the Pal-500T sample; however, these reflections disappeared after heat-treatment at 700 °C (Pal-700T). The loss of crystal identity in the Pal-700T sample occurs due to the total loss of coordinated water and irreversible dehydroxylation [61,72,73]. The characteristic reflections of quartz were unchanged, even after treatment at 700 °C for 4 h [61,74]. Calcite (CaCO_3_, ICDD 89-1305) was identified in the Pal-700T sample. The occurrence of calcite is related to the first stage of dolomite decomposition, which involves the nucleation and growth of CaCO_3_ particles [61,75,76,77].

The chemical compositions of the natural and heat-treated samples are listed in Table 1. It was observed that the Pal sample is mainly composed of SiO_2_, MgO and Al_2_O_3_, which confirms the presence of the clay mineral palygorskite since this is a hydrated silicate of magnesium and aluminum [78]. The heat-treated samples maintained their SiO_2_ and Al_2_O_3_ contents with increasing temperatures. At 700 °C, together with the appearance of calcite particles, as shown in the XRD, there is the nucleation and growth of MgO particles and CO_2_ release, according to the equation: CaMg(CO_3_)_2_ ⇆ CaCO_3_ + MgO + CO_2_ [75]. Such behavior explains the increase in MgO content in the Pal-700T sample. It was also observed that the other oxides varied for each calcination temperature. These results were associated with progressive loss of zeolitic water molecules and condensation of (–OH) groups in the clay crystal structure due to calcination [72].

**Figure 2:**
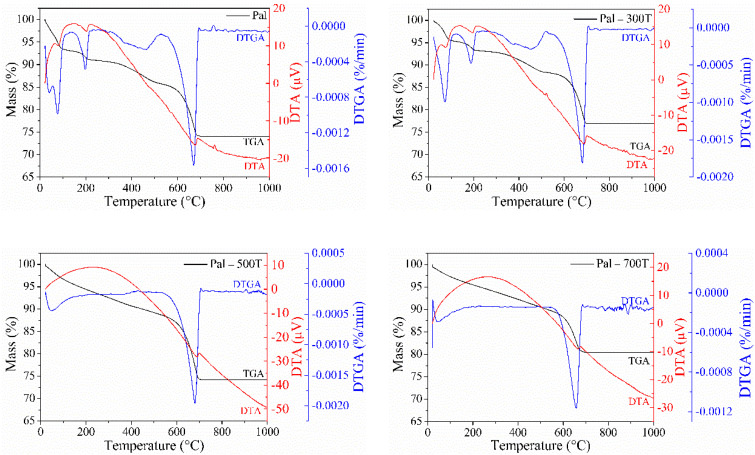
TGA-DTGA-DTA curves of palygorskite clay before and after heat treatment, obtained at a heating rate of 10 °C/min and under an air atmosphere.

The thermogravimetric (TGA), derivative thermogravimetric (DTGA) and differential thermal (DTA) curves of the natural and heat-treated palygorskite are shown in Figure 2. Four mass loss events can be identified on Pal’s TGA-DTGA curves. The first event occurred at 23–125 °C with a mass loss of 6.54% and is related to the evaporation of water physically adsorbed on the surface of the palygorskite [79]. The second event (125–230 °C) presented a mass loss of 2.46% and is related to the loss of zeolitic water molecules located in the palygorskite channels [72]. The third event observed between 230–530 °C was attributed to coordinated water loss and condensation of silanol and aluminol groups [54,80], which resulted in a mass loss of 5.26%. The last event occurred at 530–720 °C and showed a mass loss of 11.74% was caused by dolomite decomposition [81]. These four endothermic events resulted in a total mass loss of 26%. The DTA curve confirmed these events with endothermic peaks in the same temperature range as the mass losses in the TGA-DTGA curves. The peaks were attributed to: (i) evaporation of physically adsorbed water molecules [82], (ii) release of zeolitic water molecules [73], (iii) coordinated water removal, as well as the condensation of surface groups [83] and (iv) dolomite decomposition [84].

The Pal-300T sample showed mass losses and peaks (endothermic) similar to those observed in the raw palygorskite. However, it obtained a smaller mass loss in the second event (2.15%), while the Pal sample achieved a loss of 2.46%. This decrease in mass loss occurred due to the partial removal of zeolitic water during heat treatment [85]. In the Pal-500T and Pal-700T samples, there is less mass loss and the absence of peaks related to the removal of zeolitic and coordinated waters; this is a consequence of the elimination of these waters during calcination since the activation temperature was higher than the temperature of elimination of zeolitic and coordinated waters.

The specific surface area (S_BET_) and average pore diameter (D_p_) of the raw and heat-treated palygorskite samples are listed in Table 2. After heat treatment, the S_BET_ values decreased as the calcination temperature increased. Such behavior can be explained by the deformation of the palygorskite structure during dehydration and irreversible dehydroxylation, which causes pore blockage [60,61,75,86]. The D_p_ values increased as the calcination temperature increased, probably due to the formation of new and larger pores during calcination [60].

### 3.2. Adsorption Experiments

#### 3.2.1. Effect of Initial Concentration and Adsorption Isotherms

**Figure 3:**
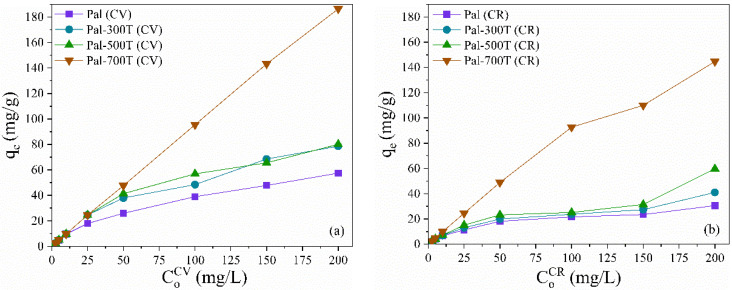
Effect of initial concentration on the adsorption capacity of natural and heat-treated palygorskite to remove (**a**) CV and (**b**) CR.

The effect of the initial concentration of crystal violet ($C_{o}^{\mathrm{CV}}$) and congo red ($C_{o}^{\mathrm{CR}}$) dyes on the adsorption capacity (q_e_) of palygorskite before and after heat treatment is shown in Figure 3a,b. Different concentrations of CV and CR (2.5 to 200 mg/L) were tested under the following experimental conditions: contact time of 360 min, pH 7, 20 mg of adsorbent at 25 °C. It is evident that the adsorption capacity of the samples increased significantly with increasing $C_{o}^{\mathrm{CV}}$ and $C_{o}^{\mathrm{CR}}$. This is because the increase in $C_{o}^{\mathrm{CV}}$ and $C_{o}^{\mathrm{CR}}$ contributed to increasing the driving force at the solid-liquid interface that overcomes the mass transfer resistance, leading to increased adsorption capacity [87,88]. The maximum CV adsorption capacity (Figure 3a) followed the order: Pal-700T (186.5 mg/g) > Pal-500T (80.1 mg/g) > Pal-300T (78.6 mg/g) > Pal (57.5 mg/g). Comparing with the Pal sample, the adsorbed amount increased by 300%, 39% and 37% to Pal-700T, Pal-500T and Pal-300T, respectively. The maximum CR adsorption capacity (Figure 3b) followed the order: Pal-700T (144.7 mg/g) > Pal-500T (59.8 mg/g) > Pal-300T (41.1 mg/g) > Pal (30.6 mg/g). Comparing the heat-treated samples with Pal, the adsorbed amount increased by 373%, 95.3% and 34.2% to Pal-700T, Pal-500T and Pal-300T, respectively. These results show that the adsorption performance of Pal for both dyes was amplified after heat treatment.

**Figure 4:**
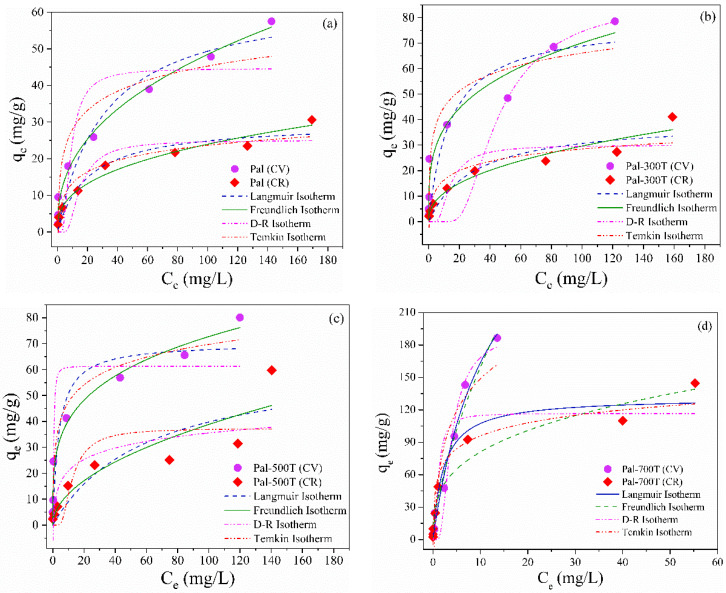
Nonlinear adsorption isotherms (Langmuir, Freundlich, D-R and Temkin) of CV and CR for (**a**) Pal, (**b**) Pal-300T, (**c**) Pal-500T and (**d**) Pal-700T.

To better understand the type of interaction that occurs between the adsorbate and the adsorbent, the experimental data of CV and CR adsorption were fitted to the nonlinear isothermal models of Langmuir, Freundlich, DR and Temkin, corresponding to Equations (3)–(6) respectively [89,90,91]:

q_e_ = (q_max_ K_L_ C_e_)/(1 + K_L_ C_e_), (3)

q_e_ = K_F_ C_e_^1/n^, (4)

q_e_ = q_D_ exp(−K_DR_ε^2^), ε = R T ln (1 + 1/C_e_), (5)

q_e_ = (RT/b_T_) lnA_T_ + (RT/b_T_) lnC_e_, (6)

where C_e_ (mg/L) is the equilibrium dye concentration and q_e_ (mg/g) is the equilibrium dye adsorbed amount. K_L_ (L/mg) is the Langmuir constant representing the activation energy in adsorption and q_max_ (mg/g), which refers to the maximum adsorption capacity. K_F_ ((mg/g)(L/mg)^1/n^) and n are the Freundlich constants, where K_F_ indicates the adsorption capacity and n corresponds to the adsorption intensity. K_DR_ (mol^2^/J^2^) and ε are the Dubinin-Radushkevich constants, while q_D_ (mg/g) is the theoretical isothermal saturation capacity. R (8314 J/mol K) is the universal gas constant and T (K) is the absolute temperature. A_T_ (L/mg) is the Temkin constant referring to the maximum binding energy and b_T_ (J/mol) is the heat of adsorption. All values were calculated from the fit of the mathematical models to the experimental data (Figure 4a–d). The results of the mathematical adjustments are shown in Table 3.

Based on the correlation coefficient values (R^2^), the Langmuir model was the one that best described the CV and CR adsorption process in the Pal-700T sample, as it exhibited R^2^ values closer to the unity. The best mathematical adjusts to the Langmuir model suggest that the adsorption of CV on Pal-700T occurs mainly via the chemisorption process, in which a monolayer of the adsorbate is deposited on the surface of the palygorskite [54,92,93]. The higher value of K_L_ means greater interaction between the adsorbent and adsorbate [94,95]; thus, Pal-700T has a greater interaction with CV molecules, while Pal has a lower interaction, which is consistent with the results obtained experimentally.

On the other hand, the result of the mathematical adjustments performed for the samples Pal, Pal-300T and Pal-500T showed that the Freundlich model was the one that best fit the experimental data of adsorption of CV and CR, as it presents R^2^ values closest to 1. Values of 1/n indicated that adsorption was favorable for all samples [94]. The Freundlich model is the best isothermal model implies that, based on its assumption, the adsorption of both dyes on Pal, Pal-300T and Pal-500T occurs on a heterogeneous surface with the formation of multiple layers [96,97].

#### 3.2.2. Effect of Contact Time and Kinetic Study

**Figure 5:**
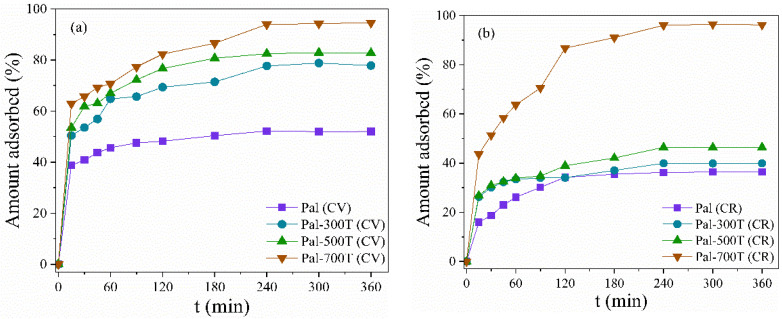
Effect of contact time on the adsorption of (**a**) CV and (**b**) CR in palygorskite before and after heat treatment.

CV and CR solutions with initial concentrations of 50 mg/L for an adsorbent amount of 20 mg at 25 °C and pH 7 were used to study the adsorption kinetics. Figure 5a shows the amount of CV adsorbed on Pal, Pal-300T, Pal-500T and Pal-700T as a function of contact time. Initially, adsorption was fast for all samples as about 40% of CV adsorption occurred within the first 30 min. Such a result is due to the active sites available on the surface of the adsorbent in the early stages [86] and the strong electrostatic interaction between the negatively charged palygorskite surface and the CV that has cationic character [98]. Over time, adsorption gradually slowed down until equilibrium was reached within 240 min. When equilibrium was reached, the adsorption capacity and CV removal value in Pal were 26.02 mg/g and 52%, respectively. While the Pal-300T, Pal-500T and Pal-700T samples removed about 78% (38.92 mg/g), 82% (41.38 mg/g) and 95% (47.3 mg/g), respectively.

Figure 5b illustrates the effect of contact time of CR adsorbed on samples before and after heat treatment. For CR, the adsorption kinetics was relatively slow, except for Pal-700T. Equilibrium was reached within 240 min with removal of 36% (18.17 mg/g), 40% (20 mg/g) and 46% (23.21 mg/g) for Pal, Pal-300T and Pal-500T, respectively. The Pal-700T sample showed rapid adsorption of CR, where in the first 30 min, about 51% of the dye had been removed. When equilibrium was reached within 240 min, the adsorption capacity of Pal-700T was 48.11 mg/g with 96% CR removed. The results revealed that Pal-700T presents excellent efficiency (greater than 90%) for removing cationic and anionic dyes.

**Figure 6:**
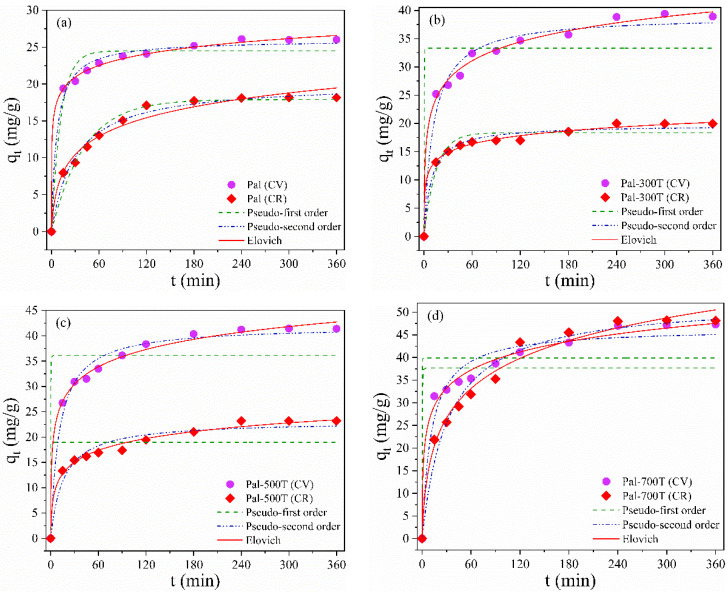
Nonlinear adsorption kinetic models (Pseudo-first order, Pseudo-second order and Elovich) of CV and CR for (**a**) Pal, (**b**) Pal-300T, (**c**) Pal-500T and (**d**) Pal-700T.

The kinetic parameters were evaluated from Pseudo-first order (Equation (7)), Pseudo-second order (Equation (8)) and Elovich (Equation (9)) models [99,100]:

q_t_ = q_e_ [1 − exp(−k_1_ t)], (7)

q_t_ = (q_e_^2^ k_2_ t)/(1 + k_2_ q_e_ t), (8)

q_t_ = α + β ln t, (9)

where q_t_ (mg/g) is the amount of dye adsorbed at time t (min) and q_e_(mg/g) is the amount of dye adsorbed at equilibrium. k_1_ (min^−1^) and k_2_ (min^−1^) are the Pseudo-first order and Pseudo-second order constants, respectively. α (mg·g^−1^/min) and β (g/mg) are the Elovich constants. Kinetic parameters were calculated from the non-linear curve fit (Figure 6a–d) and are shown in Table 4.

The Elovich model presented the highest R^2^ values; therefore, it better describes all samples’ CV and CR adsorption kinetics. The better data adjustment to the Elovich model suggests that the adsorption of CV and CR in the samples occurs by a dominant chemisorption mechanism with the adsorbents having heterogeneous surfaces [101,102].

#### 3.2.3. Effect of Temperature and Thermodynamics

**Figure 7:**
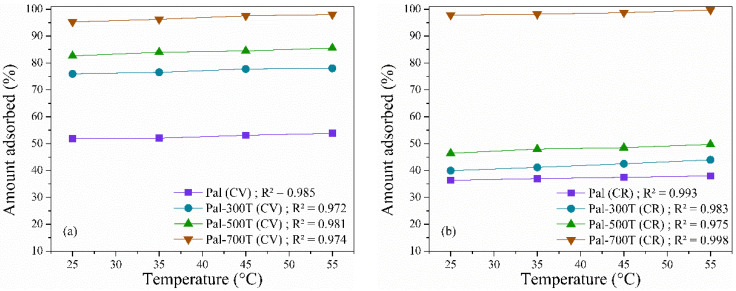
Effect of temperature on (**a**) CV and (**b**) CR adsorption on palygorskite before and after heat treatment.

The temperature analysis was carried out in the range of 25–55 °C with initial solutions of CV and CR of 50 mg/L, amount of adsorbent 20 mg at pH 7 for 360 min and the results obtained are shown in Figure 7a,b. The results showed an increase in adsorbed amounts with increasing temperature, suggesting that the adsorption of CV and CR dyes in palygorskite samples is an endothermic process [103]. This increase is a consequence of the increased mobility of CV and CR ions to the surface of the adsorbent [45,104].

The thermodynamic parameters of the adsorption process on palygorskite adsorbents were determined using the Van’t Hoff Equations (Equations (10)–(12)) [105,106]:

Ln K_d_ = (∆S/R) − (∆H/RT), (10)

∆G = −R T Ln K_d_, (11)

K_d_ = q_e_/C_e_, (12)

where K_d_ is the distribution coefficient for adsorption and ΔH, ΔS and ΔG are the enthalpy, entropy, and Gibb’s energy variations, respectively.

**Figure 8:**
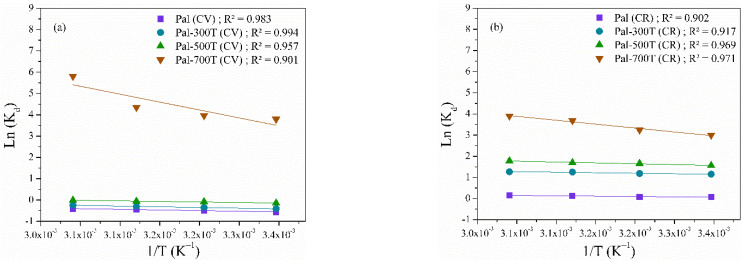
Van’t Hoff plots to determine different thermodynamic parameters in dye removal (**a**) CV and (**b**) CR.

The ΔH and ΔS values were calculated from the slope and intercept of the Ln Kd versus 1/T graph (Figure 8a,b). The values of ΔH, ΔS and ΔG are listed in Table 5. The positive values of ΔH confirmed that the adsorption of CV and CR in palygorskite samples is an endothermic process [107]. In addition, positive ΔS values indicated greater randomness at the solid-liquid interface [108] and negative ΔG values confirmed the spontaneity and viability of adsorption in the entire temperature range studied [31].

#### 3.2.4. Effect of PH Variation

**Figure 9:**
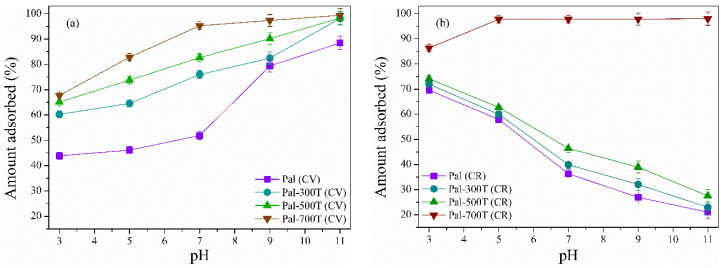
Effect of pH on the adsorption efficiency of natural and heat-treated palygorskite samples to remove (**a**) CV and (**b**) CR.

The pH is one of the main parameters in the adsorption process, as it influences the surface charge of the adsorbent [3]. The pH influence on the adsorption capacity of palygorskite samples was analyzed in the pH range of 3–11 with 20 mg of adsorbent and initial solution of CV and CR of 50 mg/L, for 360 min. All experiments were carried out at room temperature (25 °C). The pH adjustment of the dye solutions was performed using 0.1 M HCl or NH_4_OH. The amount of CV and CR adsorbed was strongly dependent on the initial pH of the solution (Figure 9a,b). The adsorbed amount of CV increased with increasing pH. The adsorbed amount of CR decreased as the pH increased (except for Pal-700T). In an acidic medium, H^+^ ions present in the solution protonate the surface of the palygorskite and the functional groups (–Si–OH) on its surface become–Si–OH^2+^ [45,53]. Therefore, the cationic CV dye adsorption is less efficient, as there is competition between the H^+^ ions and the CV molecules for the adsorption sites and the repulsive force between the surface of the positively charged adsorbent and the CV molecules decreases the removal efficiency [108]. On the other hand, in an acidic medium, the negatively charged CR molecules interact electrostatically with the positively charged palygorskite surface, presenting a higher adsorption rate [109].

In a basic medium, the OH^−^ ions promote the deprotonation of the palygorskite surface and generate –Si–O^−^ groups [110]. Therefore, the electrostatic attraction between the positively charged CV molecules and the negatively charged adsorbent surface increases, favoring adsorption [111]. In contrast, the negatively charged CR molecules compete with the OH^−^ ions and are also repelled by the negative surface of the adsorbent, which decreases the CR dye removal rate [112]. However, the adsorption of CR in the Pal-700T sample is not influenced by the pH variation, showing a removal greater than 85% at pH 3 and from pH 5, the removal is greater than 97%.

#### 3.2.5. Effect of the Amount of Adsorbent

**Figure 10:**
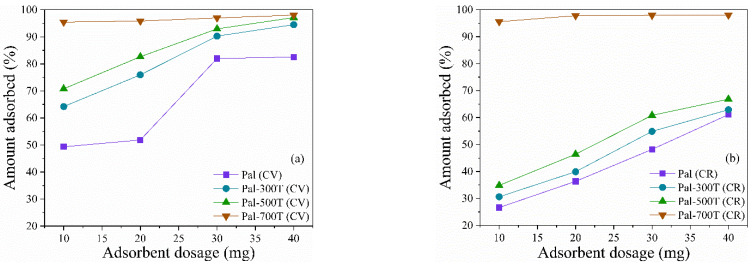
Effect of the amount of adsorbent on the adsorption of (**a**) CV and (**b**) CR on palygorskite before and after heat treatment.

Different amounts of adsorbents (10–40 mg) were used to adsorption 50 mg/L of CV and CR at 25 °C for 360 min at pH 7 (Figure 10a,b). Almost complete removal of CV and CR, both 96%, was observed with 10 mg of Pal-700T; therefore, further increase in adsorbent dosage to 40 mg has little effect on removal percentages. For the other samples, the adsorbed amount increases considerably when the mass of the adsorbent increases from 10 to 40 mg; this can be attributed to the increase in the adsorption sites available for the removal of CV and CR [113,114]. With 40 mg of adsorbent, the CV removal rate was 82.5%, 94.6%, 97.1% and 98% for Pal, Pal-300T, Pal-500T and Pal-700T, respectively. For CR, the removal rate was 61.1%, 62.9%, 66.8% and 98%, for Pal, Pal-300T, Pal-500T and Pal-700T, respectively.

### 3.3. FTIR before and after Adsorption

**Figure 11:**
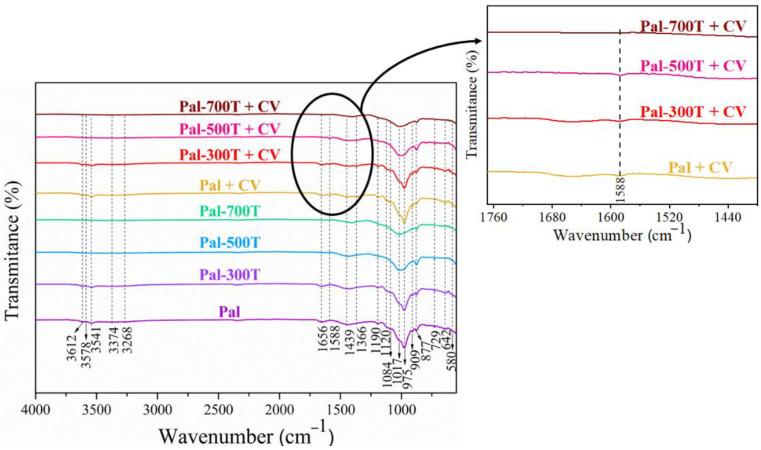
FTIR spectra of samples before and after CV adsorption.

**Figure 12:**
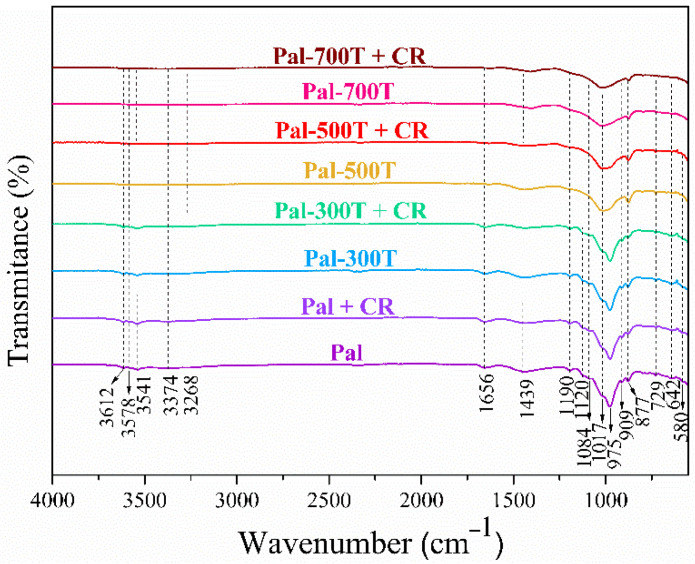
FTIR spectra of samples before and after CR adsorption.

The FTIR spectra of the samples before and after CV and CR adsorption are shown in Figure 11 and Figure 12. Bands at 3612 and 3578 cm^−1^ were identified in the spectrum of the Pal sample. Such bands are characteristic of clay minerals, the first being attributed to the Al–OH–Al elongation vibration and the second associated with the elongation vibration of the Al–Fe^3+^–OH or Al–Mg–OH bonds [115,116,117,118]. The four bands at 3541, 3374, 3268 and 1656 cm^−1^ are related to the zeolitic and coordinate waters present in the palygorskite structure [106,119]. The characteristic bands of dolomite appear at 1439 and 729 cm^−1^, related to the asymmetrical elongation of CO_3_ and the vibrations of CO_3_, respectively [120,121]. This result agrees with the XRD pattern and chemical analysis presented above, which confirm the presence of dolomite. The bands located between 1190 and 975 cm^−1^ correspond to the elongation of the Si–O bonds. The bands at 1190 and 642 cm^−1^ refer to the asymmetric and symmetrical stretching of the Si–O–Si bonds, considered fingerprints of the palygorskite [122,123]. The band at 909 cm^−1^ is related to the dioctahedral character of the palygorskite, being attributed to Al–OH–Al deformation [124,125]. The bands at 877 and 580 cm^−1^ correspond to the flexural vibration mode of the Al–Fe–OH bond [124] and Si-O strain vibration [123,126], respectively.

After heat treatment at temperatures above 300 °C, the bands at 3612 and 3578 cm^−1^ disappeared, due to complete dehydroxylation [86]. The bands related to zeolitic and coordinate water were no longer identified in the spectra of samples calcined above 300 °C, due to losses of zeolitic and coordinate water in the palygorskite structure [54]. The bands located between 1190 and 975 cm^−1^ (1190 and 642 cm^−1^) are considered fingerprints of the palygorskite. Here, due to heat treatment, there was a slight shift in such bands. Above 300 °C, the intensity of these bands decreased, and other bands disappeared. These changes are related to the condensation of silanol and/or aluminol groups via loss of water [54,72,86]. The bands at 909 and 580 cm^−1^ disappeared after heating above 300 °C.

After CV dye adsorption (Figure 11), a new band was detected at 1588 cm^−1^. This band refers to the C=C elongation vibration of the benzene ring, which is characteristic of the CV dye [127,128]. This band suggests that removing CV dye molecules from the aqueous solution occurred by chemisorption [129]. On the other hand, no new dye-related band was observed after CR dye adsorption (Figure 12). This indicates no break or formation of new bonds after adsorption, suggesting the occurrence of physical adsorption (physisorption) in the adsorption of the CR dye [18,45].

### 3.4. Recyclability Study

**Figure 13:**
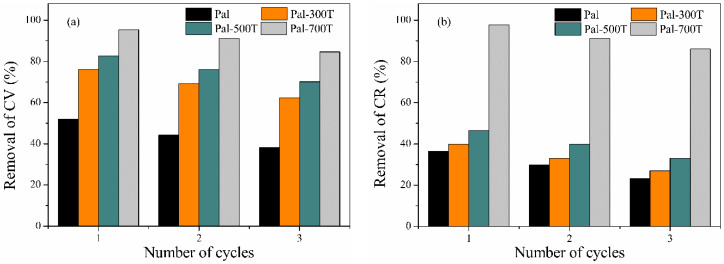
Reusability of palygorskite before and after heat treatment for three cycles to remove the dyes (**a**) crystal violet and (**b**) congo red.

To assess the recyclability of the adsorbents, 60 mL of ethanol was used to regenerate the adsorption sites. After desorption, the regenerated adsorbent was reused for three cycles and the results are shown in Figure 13a,b. Figure 13a shows the effect of regeneration on the adsorption capacity of samples for CV removal. After three cycles, the decrease in CV removal efficiency was less than 15% for all samples, going from 52%, 76%, 82% and 96% to 38%, 62%, 70% and 85% for Pal, Pal-300T, Pal-500T and Pal-700T, respectively. Figure 13b shows the effect of regeneration on the adsorption capacity of samples for CR removal. After three cycles, the decrease in CR removal efficiency was also less than 15% for all samples, going from 36%, 40%, 46% and 98% to 23%, 27%, 33% and 86% for Pal, Pal-300T, Pal-500T and Pal-700T, respectively. The above results indicate that adsorbents derived from palygorskite heat-treated have a good regeneration capacity and can be used repeatedly for the adsorption of CV and CR dyes.

## 4. Conclusions

The adsorption of CV and CR dyes on natural and calcined palygorskite at different temperatures were successfully investigated. The heat treatment promoted significant changes in the palygorskite structure. At 700 °C, the crystal structure collapsed, resulting in a smaller surface area and the formation of new and larger pores. The heat treatment increased the adsorption capacity of palygorskite. The adsorption kinetic data were better fitted to the Elovich model. The adsorption isotherms of the Pal, Pal-300T and Pal-500T samples fit well with the Freundlich isothermal model, while the Pal-700T isotherms fit better to the Langmuir isothermal model. The Pal-700T sample showed the best adsorptive performance, with a maximum adsorption capacity for CV and CR of 186.5 mg/g and 144.7 mg/g, respectively. The results revealed that Pal-700T has good adsorption affinity for cationic and anionic dyes. According to the thermodynamic results, the adsorption of CV and CR in the samples was spontaneous, favorable, and endothermic. Thus, adsorbents derived from Brazilian palygorskite proved to be promising candidates for the removal of cationic and anionic dyes from water, as they are low cost, non-toxic, ecologically correct and do not require expensive equipment to obtain them.

## Figures and Tables

**Table 1:** Chemical composition (wt %) of natural and heat-treated palygorskite samples (300, 500 and 700 °C). The margin of error was 3%.

	Sample	Pal	Pal-300T	Pal-500T	Pal-700T
Oxides (%)					
SiO_2_		52.78	51.80	52.72	52.02
MgO		13.91	14.54	14.43	15.27
Al_2_O_3_		13.48	12.70	12.89	12.92
CaO		11.92	12.77	12.11	12.09
Fe_2_O_3_		5.29	5.56	5.33	5.25
K_2_O		0.90	0.93	0.94	0.96
Other Oxides		1.72	1.70	1.58	1.49

**Table 2:** Specific surface area (S_BET_) and average pore diameter (D_p_) of palygorskite before and after heat treatment.

Sample	Specific Surface Area (m^2^/g)	Average Pore Diameter (nm)
Pal	80.4	14.3
Pal-300T	79.8	14.4
Pal-500T	67.6	16.6
Pal-700T	63.2	18.1

**Table 3:** Langmuir, Freundlich, D-R and Temkin isothermal parameters for CV and CR adsorption.

Sample	Dye	Model		
		Langmuir Isotherm		
		q_max_ (mg/g)	K_L_ (L/mg)	R^2^
Pal	CV CR	64.7 29.9	0.03 0.05	0.93 0.94
Pal-300T	CV CR	78.7 39.7	0.07 0.03	0.83 0.88
Pal-500T	CV CR	70.3 63.9	0.26 0.02	0.88 0.75
Pal-700T	CV CR	189.3 136.1	0.87 0.46	0.98 0.96
		Freundlich Isotherm		
		1/n	K_F_ (mg/g)(L/mg)^1/n^	R^2^
Pal	CV CR	0.26 0.42	11.0 4.1	0.99 0.97
Pal-300T	CV CR	0.26 0.43	19.1 4.2	0.95 0.94
Pal-500T	CV CR	0.28 0.51	22.4 4.9	0.97 0.81
Pal-700T	CV CR	0.65 0.71	35.5 39.1	0.97 0.94
		D-R Isotherm		
		q_D_ (mg/g)	K_DR_ (mol^2^/J^2^)	R^2^
Pal	CV CR	44.6 25.1	1.1 × 10^−5^ 2.9 × 10^−5^	0.79 0.82
Pal-300T	CV CR	61.3 29.9	2.6 × 10^−4^ 2.6 × 10^−5^	0.57 0.75
Pal-500T	CV CR	87.3 37.4	1.2 × 10^−7^ 2.1 × 10^−5^	0.84 0.61
Pal-700T	CV CR	191.5 116.7	2.3 × 10^−6^ 2.9 × 10^−7^	0.96 0.91
		Temkin Isotherm		
		b_T_ (J/mol)	A_T_ (L/mg)	R^2^
Pal	CV CR	329.6 551.3	4.2 1.9	0.89 0.93
Pal-300T	CV CR	267.9 468.2	12.8 2.2	0.91 0.84
Pal-500T	CV CR	255.2 389.2	13.2 2.7	0.95 0.66
Pal-700T	CV CR	57.6 143.1	25.5 3.2	0.90 0.94

**Table 4:** Pseudo-first order, Pseudo-second order and Elovich kinetic parameters for CV and CR adsorption.

Sample	Dye	Model			
		Pseudo-first order			
		q_exp_ (mg/g)	q_cal_ (mg/g)	k_1_ (min^−1^)	R^2^
Pal	CV CR	26 18.2	24.5 20.1	0.08 0.02	0.95 0.97
Pal-300T	CV CR	38.9 20	33.3 18.4	4.23 0.07	0.79 0.94
Pal-500T	CV CR	41.4 23.2	36.2 18.9	5.10 2.04	0.81 0.71
Pal-700T	CV CR	47.3 48.1	39.9 37.7	8.04 2.68	0.79 0.54
		Pseudo-second order			
		q_exp_ (mg/g)	q_cal_ (mg/g)	k_2_ (g/(mg min))	R^2^
Pal	CV CR	26 18.2	25.9 17.9	0.001 0.002	0.98 0.98
Pal-300T	CV CR	38.9 20	39.0 19.7	0.002 0.006	0.97 0.98
Pal-500T	CV CR	41.4 23.2	41.9 23.1	0.002 0.003	0.98 0.95
Pal-700T	CV CR	47.3 48.1	46.4 52.6	0.002 0.005	0.96 0.97
		Elovich			
			α (mg·g^−1^/min)	β (g/mg)	R^2^
Pal	CV CR		7.7 1.9	0.4 0.3	0.99 0.99
Pal-300T	CV CR		49.3 5.2	0.2 0.5	0.99 0.99
Pal-500T	CV CR		61.9 9.6	0.2 0.3	0.99 0.99
Pal-700T	CV CR		78.1 4.8	0.2 0.1	0.99 0.99

**Table 5:** Thermodynamic parameters for CV and CR adsorption in palygorskite before and after heat treatment at different temperatures.

Sample	Dye	∆G (kJ/mol)				∆H (kJ/mol)	ΔS (J/K mol)
		298 K	308 K	318 K	328 K		
Pal	CV	−1.41	−1.56	−1.69	−1.83	0.49	1.11
Pal-300T	CV	−0.15	−0.20	−0.29	−0.35	0.54	1.39
Pal-500T	CV	−0.66	−0.79	−0.90	−1.01	0.41	1.24
Pal-700T	CV	−9.40	−10.16	−11.49	−15.83	6.19	24.32
Pal	CR	−0.17	−0.20	−0.32	−0.41	0.27	0.97
Pal-300T	CR	−2.85	−3.02	−3.31	−3.45	0.41	2.53
Pal-500T	CR	−3.87	−4.23	−4.47	−4.83	0.68	3.85
Pal-700T	CR	−7.43	−8.29	−9.76	−10.64	3.08	13.31

## Data Availability

Not applicable.
